# Degree of Preservation of Neurovascular Bundles in Radical Prostatectomy and Recurrence of Prostate Cancer

**DOI:** 10.1016/j.euros.2021.06.005

**Published:** 2021-06-19

**Authors:** Elin Axén, Rebecka Arnsrud Godtman, Anders Bjartell, Stefan Carlsson, Eva Haglind, Jonas Hugosson, Anna Lantz, Marianne Månsson, Gunnar Steineck, Peter Wiklund, Johan Stranne

**Affiliations:** a Department of Urology, Institute of Clinical Sciences, Sahlgrenska Academy, University of Gothenburg, Gothenburg, Sweden; b Department of Urology, Sahlgrenska University Hospital, Region Västra Götaland, Gothenburg, Sweden; c Department of Urology, Skåne University Hospital, Malmö, Sweden; d Department of Molecular Medicine and Surgery, Section of Urology, Karolinska Institute, Stockholm, Sweden; e Department of Pelvic Cancer, Karolinska University Hospital, Stockholm, Sweden; f Department of Surgery, Institute of Clinical Sciences, Sahlgrenska Academy, University of Gothenburg, Scandinavian Surgical Outcomes Research Group, Gothenburg, Sweden; g Department of Surgery, Sahlgrenska University Hospital/Östra, Region Västra Götaland, Gothenburg, Sweden; h Department of Medical Epidemiology and Biostatistics, Karolinska Institute, Stockholm, Sweden; i Department of Oncology, Sahlgrenska Academy, University of Gothenburg, Gothenburg, Sweden; j Icahn School of Medicine at Mount Sinai Health System, New York, NY, USA

**Keywords:** Nerve-sparing, Prostate cancer, Radical prostatectomy, Recurrence

## Abstract

**Background:**

Reports on possible benefits for continence with nerve-sparing (NS) radical prostatectomy have expanded the indications beyond preservation of erectile function. It is unclear whether NS surgery affects oncological outcomes.

**Objective:**

To determine whether the degree of NS during radical prostatectomy influences oncological outcomes.

**Design, setting, and participants:**

Of 4003 patients enrolled in a prospective, controlled trial comparing open and robotic radical prostatectomy during 2008–2011, we evaluated 2401 patients who received robotic radical prostatectomy at seven Swedish centres. Patients were followed for 8 yr.

**Outcome measurements and statistical analysis:**

Data for recurrence and positive surgical margin status were assessed using validated patient questionnaires, patient interviews, and clinical record forms before and at 3, 12, and 24 mo and 6 and 8 yr after surgery. Cox and logistic regressions were used to model the effect on recurrence and positive surgical margins (PSM), respectively.

**Results and limitations:**

A total of 481 men had PSM and 467 experienced recurrence during follow-up. Median follow-up for men without recurrence was 6.6 yr. There were no statistically significant differences in recurrence rate between degrees of NS. The PSM rate was significantly higher with a higher degree of NS: interfascial NS, odds ratio (OR) 2.32 (95% confidence interval [CI] 1.69–3.16); intrafascial NS, OR 3.23 (95% CI 2.17–4.80). Recurrence rates were higher for patients with pT2 disease and PSM (hazard ratio [HR] 3.32, 95% CI 2.43–4.53) than for patients with pT3 disease without PSM (HR 2.08, 95% CI 1.66–2.62). The lack of central review of pathological specimens is a limitation.

**Conclusions:**

A higher degree of NS significantly increased the risk of PSM but did not significantly increase the risk of cancer recurrence. Combined with the known functional benefits of NS surgery, these results underscore the need to identify an individualised balance.

**Patient summary:**

In this report we looked at the effect of a nerve-sparing approach during removal of the prostate on cancer outcomes for patients having robot-assisted surgery at seven Swedish hospitals. We found that a high degree of nerve-sparing increased the rate of cancer positivity at the margins of surgical specimens and that positive surgical margins increased the risk of recurrence of prostate cancer.

## Take Home Message

Nerve-sparing radical prostatectomy increases the rate of positive surgical margins. Although no direct effect on recurrence is evident, this is important to address in mutual decision-making with the patient.

## 1 Introduction

Nerve-sparing (NS) radical prostatectomy was first introduced as a means to preserve erectile function after radical prostatectomy, but in recent years it has been shown that an NS technique is associated with better continence rates as well, regardless of erectile function [1], [2]. As incontinence is a common side effect of radical prostatectomy with a severe impact on quality of life, this has widened the indication for NS surgery, making more patients potential NS candidates [3]. In NS surgery the dissection planes are closer to the prostate than in the non–nerve-sparing technique, which poses a potential risk of leaving tumour tissue behind and not achieving radical resection.

The primary aim of radical prostatectomy is cure. Established predictors of recurrent disease include factors related to tumour biology, such as preoperative prostate-specific antigen (PSA), pathological stage, differentiation, and lymph node involvement, as well as procedure-related factors such as the rate of positive surgical margins (PSM) [4], [5], [6]. In previous analyses of the LAPPRO study, we observed differences in PSM patterns between robot-assisted laparoscopic prostatectomy (RALP) and open surgery [7]. The literature includes a number of reports on association between the NS degree and the rate of recurrent disease. Most studies did not find that a higher NS degree increases the risk of recurrence, but the state of the evidence is unclear as most studies are single-centre retrospective studies and are either small and/or have crude categories regarding the NS degree [8], [9], [10], [11], [12], [13], [14]. To the best of our knowledge, the only large multicentre study is a retrospective register-based study [15].

In this large, prospective cohort, we studied the influence of the NS degree during radical prostatectomy on oncological outcomes, adjusted for tumour characteristics and surgeon variability, with up to 8 yr of follow-up. The primary endpoint is recurrence, defined as biochemical recurrence (BCR) and/or a need for secondary treatment, while PSM is a secondary outcome.

## 2 Patients and methods

### 2.1 Overview

LAPPRO is a prospective, controlled, multicentre study that was originally designed to evaluate differences in continence at 12 mo after surgery between open prostatectomy and RALP. Patients were included between 2008 and 2011 at 14 Swedish urological centres; seven centres performed robotic and seven performed open surgery. The inclusion criteria at baseline were age at surgery <75 yr, PSA <20 ng/ml, clinical tumour stage <T4, and no clinical signs of distant metastasis. The study was approved by the regional ethical review board in Gothenburg (approval number 277-07) and registered in the Current Controlled Trials database (ISRCTN06393679) [16].

### 2.2 Patients

The present study is restricted to patients in LAPPRO who underwent RALP. In previous studies, we observed differences in the patterns of PSM and BCR between RALP and open surgery, with more homogeneous results for the RALP group [7]. The distribution of the NS degree differs considerably between the two techniques [17], posing potential statistical difficulties. To avoid this, we chose to analyse the most common technique. The exclusion criteria were measurable PSA at first postoperative measurement and adjuvant radiotherapy, defined as radiotherapy within 6 mo of surgery in the absence of elevated PSA or protocol violations.

### 2.3 Data collection

Preoperative characteristics and details of the surgery and PSA values and subsequent treatments were collected in case record forms at inclusion, time of surgery, and 3, 12, and 24 mo after surgery. In addition, patient questionnaires at baseline and 12 and 24 mo and 8 yr after surgery were collected by a neutral third-party secretarial service. At 6 yr, a telephone interview was conducted by research nurses. The study design is described in detail in a previous publication [18]. The NS degree was recorded separately for each lobe by the surgeon in one of four categories (intrafascial NS, interfascial NS, semi-NS, non-NS), with semi-NS defined as a dissection plane between interfascial and wide excision. The protocol did not specify indications for NS; the decision was made at the surgeon’s discretion. Multiparametric magnetic resonance imaging (mpMRI) was not routinely performed and no data for mpMRI results were collected. Surgical specimens were reviewed by local pathologists and data from the original pathology reports were entered into the case record form by the individual surgeons at each site.

### 2.4 Statistical analysis

The NS degree was categorised in four levels according to the side with the lowest NS degree, on the assumption that the side with less NS is the target tumour side. The primary endpoint was biochemical recurrence, defined as PSA >0.25 ng/ml or treatment for recurrent disease for patients with PSA ≤0.25 ng/ml at the first postoperative measurement at 6–12 wk. We used Cox proportional-hazards regression models to estimate the hazard ratio (HR) with 95% confidence interval (CI) for recurrence between the three NS groups and the non-NS group. Since the exact time of an event is not known, only that it occurs within an interval (where the left point of the interval is the last follow-up before recurrence and the right point is the first date of PSA >0.25 ng/ml or the date of treatment for recurrent disease, depending on which occurs first), we used the midpoint of the interval as the event time. The results using this approach were compared with an interval-censored approach, and the differences were negligible. No variable selection was performed, but adjustments were made with predefined confounders in two steps, with age, preoperative PSA, prostate weight, prostatectomy grading, and pathological stage in the first step, and surgeon annual volume and prior experience added in the second step. Subgroup analysis was conducted for prespecified risk groups according to the D’Amico classification: low risk, PSA ≤10 ng/ml, Gleason score ≤6, and cT1 stage; intermediate risk, PSA 10–20 ng/ml, Gleason score 7, or cT2 stage; and high risk, PSA <20 ng/ml, Gleason score ≥8, or ≥cT3 stage. PSM, defined as tumour cells on the inked margin of the specimen [19], was considered as an effect modifier rather than a confounder. Stratification by surgical margin status was applied to account for its effect. Post hoc analysis of PSM as a secondary endpoint was carried out to further explore the results obtained from stratified analysis. We used a logistic regression model to estimate the odds ratio (OR), with the same adjustments as above and PSM as the outcome. To assess whether the relationship between surgical margin status and recurrence was affected by pathological stage, the recurrence rate was analysed for each pathological stage, with or without PSM. The dependence on surgeon was handled via a so-called robust sandwich estimator of the variance. The level of statistical significance was set at 5%. All analyses are based on cases with complete data.

## 3 Results

**Fig. 1:**
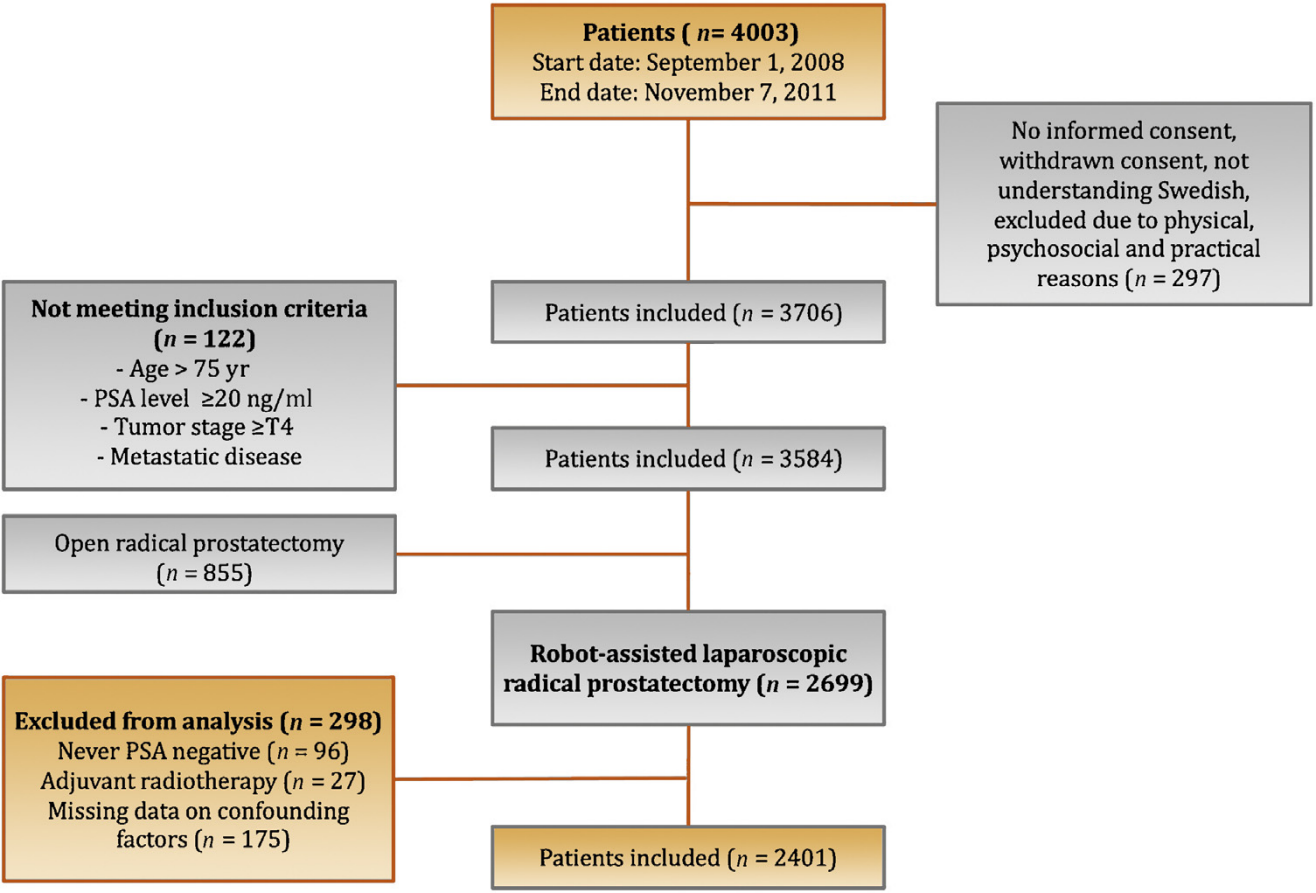
Flow chart of patient inclusion in the study. PSA = prostate-specific antigen.

Out of 4003 patients included in LAPPRO, 2401 were evaluable for the present analysis (Fig. 1). In our cohort of 2401 patients, 467 experienced recurrence on the basis of information on secondary treatment (radiotherapy in 210, BCR in 198, androgen deprivation therapy in 44, and other treatment in 15). The median follow-up for patients without recurrence was 6.6 yr (interquartile range 5.7–7.2).

Patient, tumour, and surgeon characteristics are shown in Table 1. Patients in the non-NS group had higher PSA, a higher proportion of poorly differentiated tumours, and higher pathological stage than patients receiving NS surgery. The proportion of patients with D’Amico high-risk disease according to preoperative data was highest in the non-NS group.

**Table 1:** Patient, tumour, and surgeon characteristics according to the degree of nerve-sparing in radical prostatectomy

Variable	Degree of nerve-sparing			
	None	Semi	Interfascial	Intrafascial
Patients (*n*)	1003	662	631	105
Median preoperative PSA, ng/ml (IQR)	6.4 (4.8–9.2)	6.0 (4.4–8.5)	5.4 (4.2–7.5)	5.4 (4.2–7.8)
Median age at surgery, yr (IQR)	65.8 (61.3, 68.9)	63.3 (58.7, 66.5)	61.6 (56.4, 65.3)	59.4 (54.8, 63.2)
ISUP grade in surgical specimen, *n* (%)				
Grade 1	242 (24.1)	269 (40.6)	302 (47.9)	55 (52.4)
Grade 2	449 (44.8)	280 (42.3)	257 (40.7)	39 (37.1)
Grade 3	216 (21.5)	87 (13.1)	57 (9.0)	7 (6.7)
Grade ≥4	96 (9.6)	26 (3.9)	15 (2.4)	4 (3.8)
Pathological tumour stage, *n* (%)				
pT2	615 (61.3)	507 (76.6)	552 (87.5)	92 (87.6)
≥pT3	388 (38.7)	155 (23.4)	79 (12.5)	13 (12.4)
Median prostate weight, g (IQR)	43.4 (36.0–55.0)	41.0 (33.5–51.1)	41.7 (33.5–52.0)	38.0 (31.0–47.0)
Prior procedures performed by OpS, *n* (%)				
<100	360 (35.9)	206 (31.1)	166 (26.3)	22 (21.0)
≥100	643 (64.1)	456 (68.9)	465 (73.7)	83 (79.0)
Annual procedures performed by OpS, *n* (%)				
<50	507 (50.5)	314 (47.4)	168 (26.6)	63 (60.0)
≥50	496 (49.5)	348 (52.6)	463 (73.4)	42 (40.0)
Preoperative D’Amico risk group, *n* (%)				
Low	113 (11.3)	184 (27.8)	357 (56.6)	66 (62.9)
Medium	748 (74.6)	448 (67.7)	271 (42.9)	38 (36.2)
High	142 (14.2)	30 (4.5)	3 (0.5)	1 (1.0)

IQR = interquartile range; ISUP = International Society of Urological Pathology; OpS = operating surgeon; PSA = prostate-specific antigen.

**Fig. 2:**
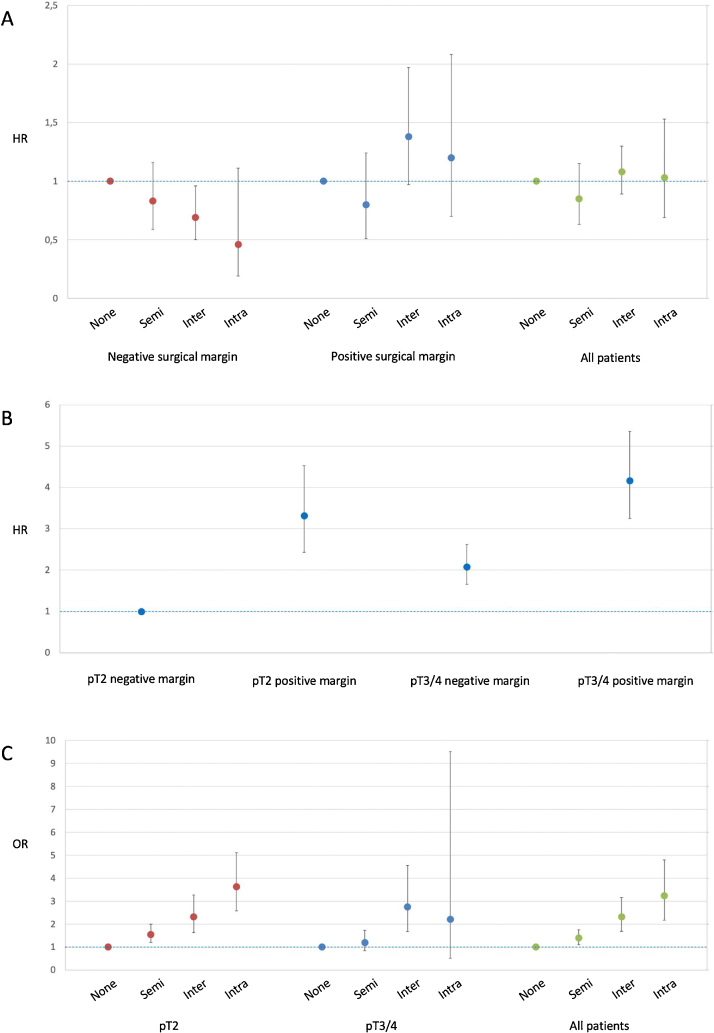
(A) Hazard ratio (HR) with 95% confidence interval for recurrence by degree of nerve-sparing for all patients and patients with negative versus positive surgical margins, adjusted patient age, preoperative prostate-specific antigen, International Society of Urological Pathology grade in surgical specimen, pathological stage, prostate weight and surgeon prior experience and annual volume. (B) HR with 95% confidence interval for recurrence according to pathological stage with versus without positive margins, adjusted for patient age, preoperative prostate-specific antigen, International Society of Urological Pathology grade in surgical specimen, prostate weight, and surgeon prior experience and annual volume. (C) Odds ratio (OR) with 95% confidence interval for positive surgical margins for different degrees of nerve-sparing according to pathological stage, adjusted for patient age, preoperative prostate-specific antigen, International Society of Urological Pathology grade in surgical specimen, prostate weight, and surgeon prior experience and annual volume. Intra = intrafascial nerve-sparing surgery; Inter = interfascial nerve-sparing surgery; None = no nerve-sparing; Semi = semi–nerve-sparing surgery.

The recurrence rate was 24.9% in the non-NS, 15.9% in the semi-NS, 15.2% in the interfacial NS, and 15.2% in the intrafascial NS group. The HRs for recurrence were significantly lower for any degree of NS when compared to non-NS in unadjusted analysis. After adjustment for patient age and tumour characteristics, there were no significant differences between the groups. The results were largely unchanged by additional adjustment for surgeon experience (Table 2). When stratified by surgical margin status, point estimates for recurrence for interfascial and intrafascial NS differed between the groups with positive and negative surgical margin status. However, the CIs were wide and the effects were not significant (Fig. 2A, Supplementary Table 1). The recurrence rate was higher with positive than with negative surgical margin status for both pT2 stage and combined pT3/4 stage, with the recurrence rate for pT2 with PSM exceeding the rate for pT3/4 with negative margins (Fig. 2B, Supplementary Table 1).

**Table 2:** Hazard ratio for recurrence of prostate cancer according to the degree of nerve-sparing in radical prostatectomy

	Hazard ratio (95% confidence interval)		
	Unadjusted	Adjusted A a	Adjusted B b
Degree of nerve-sparing			
None	1.00	1.00	1.00
Semi	0.61 (0.42–0.89)	0.85 (0.62–1.17)	0.85 (0.63–1.15)
Interfascial	0.58 (0.45–0.74)	1.11 (0.88–1.39)	1.08 (0.89–1.30)
Intrafascial	0.56 (0.39–0.81)	1.02 (0.67–1.57)	1.03 (0.69–1.53)
Preoperative PSA	–	1.06 (1.02–1.10)	1.06 (1.02–1.10)
Age at surgery	–	1.02 (1.00–1.03)	1.02 (1.00–1.03)
ISUP grade in surgical specimen			
Grade 1	–	1.00	1.00
Grade 2	–	2.19 (1.68–2.86)	2.20 (1.69–2.87)
Grade ≥3	–	4.33 (2.93–6.41)	4.35 (2.94–6.44)
Pathological tumour stage			
pT2	–	1.00	1.00
pT3/4	–	1.93 (1.59–2.34)	1.92 (1.59–2.32)
Prostate weight	–	0.99 (0.98–1.00)	0.99 (0.98–1.00)
Prior procedures performed by OpS			
<100	–	–	1.00
≥100	–	–	1.04 (0.75–1.43)
Annual procedures performed by OpS			
<50	–	–	1.00
≥50	–	–	1.09 (0.82–1.45)

ISUP = International Society of Urological Pathology; OpS = operating surgeon.

a Adjusted for age at surgery, preoperative prostate-specific antigen, ISUP grade in surgical specimen, pathological tumour stage, and prostate weight.

b Adjusted for age at surgery, preoperative prostate-specific antigen, ISUP grade in surgical specimen, pathological tumour stage, prostate weight, surgeon prior experience, and surgeon annual volume.

The PSM frequency was 20% overall (pT2 16.5%, pT3/4 29.9%). The PSM rate increased with the NS degree, and the ORs were significantly higher for any degree of NS versus non-NS in adjusted analyses. When patients were subdivided by pathological stage, the same linear relationship persisted for pT2 but was less pronounced for pT3/4 (Fig. 2C and Table 3).

**Table 3:** Odds ratio for positive surgical margins according to the degree of nerve-sparing in radical prostatectomy

	Degree of nerve-sparing			
	None	Semi	Interfascial	Intrafascial
**pT2 disease**				
Patients with PSM, *n*/*N* (%)	72/615 (11.7)	81/507 (16.0)	111/552 (20.1)	27/92 (29.3)
Unadjusted OR (95% CI)	1.00	1.43 (1.07–1.93)	1.90 (1.28–2.81)	3.13 (2.12–4.63)
Adjusted OR (95% CI) a	1.00	1.54 (1.18–2.00)	2.24 (1.60–3.15)	3.71 (2.62–5.25)
Adjusted OR (95% CI) b	1.00	1.55 (1.20–2.00)	2.31 (1.63–3.27)	3.63 (2.58–5.10)
**pT3/4 disease**				
Patients with PSM, *n*/*N* (%)	106/388 (27.3)	45/155 (29.0)	34/79 (43.0)	5/13 (38.5)
Unadjusted OR (95% CI)	1.00	1.09 (0.76–1.56)	2.01 (1.31–3.08)	1.66 (0.39–7.08)
Adjusted OR (95% CI) a	1.00	1.20 (0.83–1.74)	2.54 (1.63–3.96)	2.25 (0.47–10.79)
Adjusted OR (95% CI) b	1.00	1.19 (0.83–1.72)	2.75 (1.67–4.55)	2.20 (0.51–9.52)
**All patients**				
Patients with PSM, *n*/*N* (%)	178/1003 (17.7)	126/662 (19.0)	145/631 (23.0)	32/105 (30.5)
Unadjusted OR (95% CI)	1.00	1.09 (0.86–1.38)	1.38 (1.03–1.85)	2.03 (1.36–3.03)
Adjusted OR (95% CI) a	1.00	1.39 (1.10–1.76)	2.23 (1.64–3.03)	3.31 (2.14–5.14)
Adjusted OR (95% CI) b	1.00	1.39 (1.11–1.75)	2.32 (1.69–3.16)	3.23 (2.17–4.80)

CI = confidence interval; OR = odds ratio; PSM = positive surgical margins.

a Adjusted for age at surgery, preoperative prostate-specific antigen, International Society of Urological Pathology grade in surgical specimen, pathological tumour stage, and prostate weight.

b Adjusted for age at surgery, preoperative prostate-specific antigen, International Society of Urological Pathology grade in surgical specimen, pathological tumour stage, prostate weight, surgeon prior experience, and surgeon annual volume.

Subgroup analysis of the recurrence rate for D’Amico low and medium risk showed no significant effects of NS. Analysis of D’Amico high risk was not possible as very few patients in this group had higher NS degrees (Table 1 and Supplementary Table 2).

## 4 Discussion

In this large, controlled, prospective study we could not demonstrate that a higher NS degree in LARP increased the risk of prostate cancer recurrence. The risk of recurrence is highly dependent on tumour biology, for which a number of clinical risk factors are proxies. The best-documented risk factors for recurrence at present are PSA, histological grade, and tumour stage [4], [5]. Preoperative risk assessment influences decision-making regarding the NS degree, whereby patients at the highest risk of recurrence more frequently undergo non-NS surgery [15], [20]. When stratified by surgical margin status, point estimates for recurrence in the negative surgical margin group decreased with increasing NS degree. Even though the effects were not statistically significant, this suggests a high level of residual confounding. Long-term prognosis after radical prostatectomy has considerable variability and nomograms based on clinical data provide tools for risk assessment [4], [5]. It has been shown that addition of genetic markers to such nomograms independently affect prognosis. When modelled in receiver operating characteristic curves, inclusion of genetic markers in prognostic models based on established clinical risk scores increased the area under the curve by approximately 10 percentage points [21], [22]. This supports the notion that adjustment for clinical risk factors, as in our study, is insufficient to control for differences in prognosis related to tumour biology.

PSM is a predictor of recurrence that depends on both the tumour and the surgeon, but few studies have shown any effect of NS surgery on PSM rates [11], [14], [15], [23], [24]. In our study there was a linear relationship between the NS degree and the PSM rate. The effect was most pronounced in pT2 disease, for which the OR for PSM for intrafascial NS was 3.6 times higher than for non-NS surgery. It has been shown that PSM in organ-confined disease is largely avoidable and decreases with increasing surgeon experience, with rates reported as low as 4% [25]. The number of prior surgeries needed to reach a plateau in PSM rates differs markedly between studies, from 250 to 1600 cases [23], [26], [27]. Adjustment for surgeon experience had only a minor impact in our data, which can possibly be attributed to the limited range for the number of prior surgeries for participating surgeons [28]. PSM had a major impact on the recurrence rate, irrespective of pathological tumour stage. The recurrence rate was higher for pT2 with PSM (OR 3.31, 95% CI 2.42–4.53) than for pT3/4 with negative margins (OR 2.09, 95% CI 1.66–2.63). Similar findings have previously been reported [29], [30]. Preoperative mpMRI improves the prediction of adverse findings at radical prostatectomy, but the role of imaging in surgical planning remains to be established [31]. No mpMRI data were available for this cohort.

Radical prostatectomy is an oncological procedure aimed at curing prostate cancer, while efforts to minimise functional side effects are secondary objectives [3]. BCR is a surrogate marker for oncological outcome, and many patients with BCR are exposed to salvage treatment with well-known adverse effects [32], [33], [34]. Studies on postoperative quality of life are scarce, but there are data indicating that both PSM and recurrence have a significant negative effect on psychosocial wellbeing [35], [36]. Although the effect of recurrence on overall mortality is limited [32], the difference for patients with and without recurrence is having cancer in the past and in the present. Individual information on the pros and cons of NS surgery should be presented to the patient, and whether to prioritise limitation of immediate side effects or reduction of the risk of recurrence should be a mutual decision in which the patient’s preferences should be weighted heavily.

In a study of 7268 patients in 2004, Ward et al [10] found no significant difference in biochemical progression–free survival between NS surgery (unilateral or bilateral) and wide excision in multivariable analysis. These results are in agreement with our findings. By contrast, they found no difference in PSM between NS surgery and wide excision, with an adjusted OR of 0.89 (95% CI 0.79–1.01). PSM rates declined during the latter part of the study period, but were high overall (mean 38%). Their results are not directly comparable to ours because of different definitions of the degree of NS and the use of intraoperative frozen sections. By contrast, in a 2014 study of 1133 patients with pT2 disease, Røder et al [20] observed a 68% increase in PSM for NS compared to non-NS surgery. They also found that PSM significantly increased the risk of recurrence (HR 2.4, 95% CI 1.6–3.6) but, as in our study, this was not reflected in recurrence rates in relation to NS status. However, for patients with PSM the HR for recurrence was 4.2 for NS versus 1.9 for non-NS surgery, although the difference was not significant (*p* = 0.08).

The strengths of our study include the prospective design, the large sample size, and the long follow-up with a high response rate. Furthermore, we detailed prospective information on the level of NS in relation to the surgical plane, making it possible to define precise categories regarding the NS degree. Patients were not randomised regarding the degree of NS, which is a limitation. We attempted to adjust for preanalytical selection bias using postoperative histopathological data, since these are the most accurate measurements of tumour biology available. Even though the decision on NS degree is made preoperatively or intraoperatively when these data are not known, selection is according to the assumption that the characteristics reflected in preoperative data.

We defined recurrence according to PSA levels and secondary therapies. Since secondary therapies, with the exception of adjuvant radiotherapy (which we excluded), are given to treat recurrence, this approach might influence the time recorded for recurrence, but not the event itself. The lack of central review of pathological specimens is a limitation. PSM assignment is interpreter-dependent, but any systematic bias seems unlikely since local pathologists review specimens for all degrees of NS. A previous LAPPRO analysis comparing original pathology reports for a random sample of cases to results from review by two reference pathologists showed acceptable concordance for PSM (κ = 0.76) [37]. It has been shown that the PSM extent correlates with the risk of recurrence, and the lack of data on PSM length is another limitation of our study [38], [39]. Short PSM lengths might in part explain why the higher risk of PSM with NS surgery in our cohort does not translate to a higher risk of recurrence. There is also a risk of misclassification regarding pathological stage for specimens with PSM and a lack of extraprostatic tissue, possibly inflating the recurrence risk for pT2 tumours with PSM [40].

## 5 Conclusions

Our results show no statistically significant effect of the NS degree on the risk of recurrence; however, a higher NS degree significantly increased the risk of PSM, irrespective of tumour stage, and PSM in turn increased the risk of recurrence. Our results suffer from a high degree of residual confounding. In the presence of unknown confounders, only a randomised study is likely to be sufficient to resolve the issue of potential negative effects of NS surgery on recurrence. Since there are known functional benefits of NS surgery, ethical considerations limit the possibility of conducting such a study. Finding the balance between these two outcomes that best suits the individual is essential. Our results add another piece to this puzzle.

  ***Author contributions***: Elin Axén had full access to all the data in the study and takes responsibility for the integrity of the data and the accuracy of the data analysis.

  *Study concept and design*: Axén, Arnsrud Godtman, Bjartell, Carlsson, Haglind, Hugosson, Lantz, Månsson, Steineck, Wiklund, Stranne.

*Acquisition of data*: Axén, Bjartell, Carlsson, Haglind, Hugosson, Lantz, Steineck, Wiklund, Stranne.

*Analysis and interpretation of data*: Axén, Arnsrud Godtman, Bjartell, Carlsson, Haglind, Hugosson, Lantz, Månsson, Steineck, Wiklund, Stranne.

*Drafting of the manuscript*: Axén.

*Critical revision of the manuscript for important intellectual content*: Axén, Arnsrud Godtman, Bjartell, Carlsson, Haglind, Hugosson, Lantz, Månsson, Steineck, Wiklund, Stranne.

*Statistical analysis*: Axén, Månsson.

*Obtaining funding*: Haglind, Stranne.

*Administrative, technical, or material support*: None.

*Supervision*: Stranne.

*Other*: None.

  ***Financial disclosures:*** Elin Axén certifies that all conflicts of interest, including specific financial interests and relationships and affiliations relevant to the subject matter or materials discussed in the manuscript (eg, employment/affiliation, grants or funding, consultancies, honoraria, stock ownership or options, expert testimony, royalties, or patents filed, received, or pending), are the following: Johan Stranne has received lecture fees from IPSEN, Astellas, Bayer, Jansen, and Ferring, and has worked as a surgical proctor for Intuitive Surgery. Rebecka Arnsrud Godtman has received lecture fees from IPSEN and Astellas. Gunnar Steineck works as chief medical officer at AroCell AB, a company that sells a biomarker that can be used in managing prostate cancer. Eva Haglind has received research grants from all organisations and foundations listed as sponsors. The remaining authors have nothing to disclose.

  ***Funding/Support and role of the sponsor*:** The LAPPRO study was supported by research grants from the 10.13039/501100002794Swedish Cancer Society (2008/922, 2010/593, 2013/497, 2016/362, and 2019 0303), The Swedish Research Council (2012-1770 and 2015-02483), Region Västra Götaland, 10.13039/501100005754Sahlgrenska University Hospital (ALFGBG grants 13875, 146201, 4307771, and 718221; HTA–VGR 6011 agreement concerning research and education of doctors), the Mrs. Mary von Sydow Foundation, and the Anna and Edwin Berger Foundation. Part of Elin Axén’s work on this paper was supported by grants from Region Västra Götaland, Sahlgrenska University Hospital (ALFGBG grant 720421; agreement concerning research and education of doctors). The funders had no role in the design or conduct of the study, including collection, management, analysis, and interpretation of the data; and preparation, review, or approval of the manuscript.
