# Supplementary material for: Degree of Preservation of Neurovascular Bundles in Radical Prostatectomy and Recurrence of Prostate Cancer
Source: Eur Urol Open Sci. 2021 Jun 19;30:25–33. doi: 10.1016/j.euros.2021.06.005 (PMC8317882; doi:10.1016/j.euros.2021.06.005)
Supplement: Supplementary file 1 [file mmc1.docx]

| Supplementary table 1. | | | | | | | |
| --- | --- | --- | --- | --- | --- | --- | --- |
| 1. Hazard ratio (HR) for recurrence according to degree of nerve-sparing stratified on surgical margin status | | | | | | | |
|  |  | None | | Semi | Inter | | Intra |
| Negative surgical margins | Patients with recurrence, n/N (%) | 172/825 (20.8) | | 66/536 (12.3) | 39/486 (8.0) | | 4/73 (5.4) |
|  | Unadjusted, HR (95% CI) | 1.00 | | 0.56 (0.36-0.89) | 0.36 (0.27-0.48) | | 0.22 (0.09-0.56) |
|  | Adjusted A*, HR (95% CI) | 1.00 | | 0.83 (0.57-1.20) | 0.73 (0.52-1.02) | | 0.45 (0.18-1.13) |
|  | Adjusted B**, HR (95% CI) | 1.00 | | 0.83 (0.59-1.16) | 0.69 (0.50-0.96) | | 0.46 (0.19-1.11) |
|  |  |  | |  |  | |  |
| Positive surgical margins | Patients with recurrence, n/N (%) | 78/178 (43.8) | | 39/126 (30.9) | 57/145 (39.3) | | 12/32 (37.5) |
|  | Unadjusted, HR (95% CI) | 1.00 | | 0.69 (0.46-1.03) | 0.86 (0.60-1.24) | | 0.89 (0.58-1.37) |
|  | Adjusted A*, HR (95% CI) | 1.00 | | 0.80 (0.51-1.24) | 1.30 (0.88-1.91) | | 1.25 (0.71-2.21) |
|  | Adjusted B**, HR (95% CI) | 1.00 | | 0.80 (0.51-1.24) | 1.38 (0.97-1.97) | | 1.20 (0.70-2.08) |
|  |  |  | |  |  | |  |
| 1. Hazard ratio (HR) for recurrence according to pathological stage and surgical margin status | | | | | | | |
|  |  | | n/N (%) | | | Adjusted C***, HR (95% CI) | |
| pT2 | Negative surgical margins | | 147/1475 (10.0) | | | 1.00 | |
|  | Positive surgical margins | | 1475/311 (30.5) | | | 3.32 (2.43-4.53) | |
| pT3/4 | Negative surgical margins | | 311/134 (30.1) | | | 4.17 (3.25-5.36) | |
|  | Positive surgical margins | | 134/445 (51.0) | | | 2.08 (1.66-2.62) | |
|  |  | |  | | |  | |
|  | *Age at surgery, preoperative PSA, ISUP grading in surgical specimen, pathological tumour stage, prostate weight | | | | | | |
|  | ** Adjustment A and surgeon prior experience, surgeon annual volume | | | | | | |
|  | *** Age at surgery, preoperative PSA, ISUP grading in surgical specimen, prostate weight, surgeon prior experience, surgeon annual volume | | | | | | |
|  | *pT2= pathological tumour stage 2 pT3/4= pathological tumour stage 3 or 4* | | | | | | |

| Supplementary table 2. Odds ratio (OR) for positive surgical margins | | | |
| --- | --- | --- | --- |
|  |  | pT2 | pT3/4 |
| Unadjusted, OR (95%CI) | | | |
| Degree of nerve-sparing | None | 1.00 | 1.00 |
|  | Semi | 1.43 (1.07-1.93) | 1.09 (0.76-1.56) |
|  | Inter | 1.90 (1.28-2.81) | 2.01 (1.31-3.08) |
|  | Intra | 3.13 (2.12-4.63) | 1.66 (0.39-7.08) |
| Adjustment A*, OR (95%CI) | | | |
| Degree of nerve-sparing | None | 1.00 | 1.00 |
|  | Semi | 1.54 (1.18-2.00) | 1.20 (0.83-1.74) |
|  | Inter | 2.24 (1.60-3.15) | 2.54 (1.63-3.96) |
|  | Intra | 3.71 (2.62-5.25) | 2.25 (0.47-10.79) |
| Preoperative PSA |  | 1.07 (1.02-1.12) | 1.06 (1.03-1.10) |
| Age at surgery |  | 1.03(1.00-1.05) | 1.04 (1.01-1.08) |
| ISUP grade in surgical specimen | 1 |  | 1.00 () |
|  | 2 | 1.43 (1.08-1.88) | 0.56 (0.30-1.05) |
|  | >=3 | 1.25 (0.85-1.83) | 0.80 (0.45-1.41) |
| Prostate weight |  | 0.98 (0.97-0.99) | 0.99 (0.98-1.00) |
| Adjustment B**, OR (95%CI) | | | |
| Degree of nerve-sparing | None | 1.00 | 1.00 |
|  | Semi | 1.55 (1.20-2.00) | 1.19 (0.83-1.72) |
|  | Inter | 2.31 (1.63-3.27) | 2.75 (1.67-4.55) |
|  | Intra | 3.63 (2.58-5.10) | 2.20 (0.51-9.52) |
| Preoperative PSA |  | 1.07 (1.02-1.12) | 1.06 (1.03-1.10) |
| Age at surgery |  | 1.03 (1.00-1.05) | 1.04 (1.01-1.08) |
| ISUP grade in surgical specimen | 1 | 1.00 | 1.00 |
|  | 2 | 1.42 (1.07-1.89) | 0.54 (0.28-1.02) |
|  | >=3 | 1.23 (0.84-1.81) | 0.77 (0.45-1.31) |
| Prostate weight |  | 0.98 (0.97-0.99) | 1.00 (0.99-1.00) |
| No of prior procedures performed by operating surgeon | <100 | 1.00 | 1.00 |
|  | ≥100 | 1.07 (0.79-1.43) | 1.04 (0.65-1.67) |
| No of annual procedures performed by operating surgeon | <50 | 1.00 | 1.00 |
|  | ≥50 | 0.86 (0.69-1.08) | 0.73 (0.52-1.04) |

|  | *Age at surgery, preoperative PSA, ISUP grading in surgical specimen, pathological tumour stage, prostate weight |
| --- | --- |
|  | ** Adjustment A and surgeon prior experience, surgeon annual volume |
|  | *** Age at surgery, preoperative PSA, ISUP grading in surgical specimen, prostate weight, surgeon prior experience, surgeon annual volume |
|  | *pT2= pathological tumour stage 2 pT3/4= pathological tumour stage 3 or 4* |

| Supplementary table 3. Hazard ratio (HR) for recurrence according to degree of nerve-sparing for D’Amico risk groups | | | | | |
| --- | --- | --- | --- | --- | --- |
|  |  | None | Semi | Inter | Intra |
| Low risk | Patients with recurrence, n/N (%) | 16/113 (14.1) | 12/184 (6.52) | 40/357 (11.2) | 7/66 (10.6) |
|  | Unadjusted, HR (95% CI) | 1.00 | 0.48 (0.23-1.00) | 0.82 (0.60-1.12) | 0.76 (0.41-1.43) |
|  | Adjusted A*, HR (95% CI) | 1.00 | 0.50 (0.24-1.05) | 0.90 (0.60-1.34) | 0.97 (0.62-1.52) |
|  | Adjusted B**, HR (95% CI) | 1.00 | 0.51 (0.22-1.15) | 0.96 (0.66-1.40) | 0.96 (0.62-1.47) |
|  |  |  |  |  |  |
| Medium risk | Patients with recurrence, n/N (%) | 178/748 (23.7) | 80/448 (17.8) | 55/271 (20.2) | 9/38 (23.6) |
|  | Unadjusted, HR (95% CI) | 1.00 | 0.72 (0.53-0.99) | 0.83 (0.59-1.17) | 0.95 (0.61-1.49) |
|  | Adjusted A*, HR (95% CI) | 1.00 | 0.93 (0.69-1.25) | 1.29 (0.90-1.84) | 1.34 (0.85-2.13) |
|  | Adjusted B**, HR (95% CI) | 1.00 | 0.92 (0.69-1.22) | 1.27 (0.93-1.74) | 1.36 (0.87-2.11) |
|  |  |  |  |  |  |
|  | *Age at surgery, preoperative PSA, ISUP grading in surgical specimen, pathological tumour stage, prostate weight | | | | |
|  | ** Adjustment A and surgeon prior experience, surgeon annual volume | | | | |
